# Mono- and co-infections of primary porcine respiratory cells with *Bordetella bronchiseptica* and *Streptococcus suis* are not affected by the dermonecrotic toxin

**DOI:** 10.1128/iai.00366-25

**Published:** 2026-03-12

**Authors:** Désirée Schaaf, Muriel Dresen, Yenehiwot Berhanu Weldearegay, Jeannine Biermann, Susan L. Brockmeier, Wolfgang Baumgärtner, Michael Jarek, Peter Valentin-Weigand

**Affiliations:** 1 Institute for Microbiology, University of Veterinary Medicine Hannover 26556 , Hanover, Germany; 2 Virus and Prion Research Unit, National Animal Disease Center, ARS, USDA 57837 , Ames, Iowa, USA; 3 Department of Pathology, University of Veterinary Medicine Hannover 26556 , Hanover, Germany; 4 Genome Analytics (GMAK), Helmholtz Centre for Infection Research (HZI) 28336, Braunschweig, Germany; Stanford University School of Medicine, Stanford, California, USA

**Keywords:** air-liquid interface cultures, porcine precision-cut lung slices, porcine respiratory tract infection, *Streptococcus suis*, dermonecrotic toxin, *Bordetella bronchiseptica*

## Abstract

*Bordetella bronchiseptica* is a gram-negative bacterium contributing to respiratory diseases in many different animal species. In the swine population, it occurs frequently and plays a role in the Porcine Respiratory Disease Complex as well as in the pathogenesis of atrophic rhinitis. The dermonecrotic toxin (DNT) is involved in the destruction of the nasal conchae, a hallmark of atrophic rhinitis, and several studies have shown the effects of DNT on osteoblastic cells. Surprisingly, only little is known about the interactions of DNT and respiratory epithelial cells. Thus, we investigated the influence of DNT on porcine respiratory epithelial cells during mono- and co-infections *in vitro*. For this, we infected porcine precision-cut lung slices and air-liquid interface cultures with a DNT-positive *B. bronchiseptica* wild-type strain and its isogenic DNT-deficient mutant strain. For co-infection experiments, a *Streptococcus suis* serotype 2 wild-type strain was used. We evaluated cytotoxic effects and colonization of both pathogens, as well as the pro-inflammatory cytokine response of the host cells. Remarkably, DNT neither contributed to the cytotoxic effects of *B. bronchiseptica* nor did it affect bacterial colonization. Regarding the cytokine response, pro-inflammatory cytokines were expressed mainly upon infection with *B. bronchiseptica* but hardly after infection with *S. suis*, whereas co-infection with both pathogens had an amplifying effect on cytokine expression after prolonged infection, independently of DNT. Concluding, we found no evidence that DNT contributes to the early stages of infection with *B. bronchiseptica* and *S. suis* in *in vitro* models of the porcine respiratory tract.

## INTRODUCTION

*Bordetella bronchiseptica* is a small, rod-shaped, and gram-negative bacterium that is closely related to the human pathogen *Bordetella pertussis*, the causative agent of whooping cough. In contrast to its host-specific relative, *B. bronchiseptica* can cause respiratory infections of multiple etiology in many different animal species as well as in humans. In pigs, *B. bronchiseptica* is the causative agent of non-progressive atrophic rhinitis, which can, in the presence of toxigenic *Pasteurella multocida*, lead to the severe progressive form (1). Turbinate atrophy caused by *B. bronchiseptica* can be associated with the dermonecrotic toxin (DNT), a cytoplasmic, heat-labile toxin belonging to the group of cytotoxic necrotizing factors that is almost identical in *B. bronchiseptica* and *B. pertussis* (2–4). *In vitro* studies have shown that purified DNT induces tremendous morphological changes of the cytoskeleton due to DNT-mediated activation of the small GTP-binding protein Rho and other members of the Rho family (5). Moreover, it can inhibit the differentiation of osteoblastic cells (6), resulting in deformation of the nasal conchae as typically seen in atrophic rhinitis. However, to our knowledge, nothing is known about the effects of DNT on respiratory epithelial cells. Recently, the T-type voltage-gated calcium channels Ca_v_3.1 and Ca_v_3.2 were identified as DNT-binding receptors in different mouse cell lines (3), which are located on neurons, cardiac, and skeletal cells, but are also expressed by human lung epithelial cells (7).

As part of the Porcine Respiratory Disease Complex (PRDC), *B. bronchiseptica* also contributes to pneumonia in pigs and can pave the way for infection with secondary pathogens, for example, *Streptococcus suis*, as we have shown in our previous study (8). *S. suis* is a facultative pathogenic, gram-positive bacterium colonizing the upper respiratory tract of almost all pigs. However, this pathobiont can become invasive when the respiratory epithelial barrier has been damaged by a previous infection with, for example, swine influenza virus (SIV) (9, 10) and cause severe systemic diseases in piglets. Some studies have been published on interactions between *B. bronchiseptica* and *S. suis,* but it is yet unclear how *B. bronchiseptica* can predispose to infection with *S. suis*. Thus, in this study, we focused on (i) the role of DNT in interactions between the two pathogens and (ii) the following pro-inflammatory cytokine response of the host to co-infection with *B. bronchiseptica* and *S. suis*. We assumed that DNT can contribute to the cytotoxic effects and the colonization of *B. bronchiseptica*, as the latter was described in a previous *in vivo* study (11), consequently affecting also co-infection with *S. suis*. Furthermore, we expected immunomodulatory effects of a pre-infection with *B. bronchiseptica* as well as a synergistic pro-inflammatory response induced by both pathogens, as it has been described for other co-infections of the (porcine) respiratory tract (12–15).

As only a little is known about the immune response during porcine respiratory tract infection with *B. bronchiseptica*, we set out to investigate this in our *in vitro* infection models—porcine respiratory epithelial cells well-differentiated under air-liquid interface conditions (ALI cultures) and the porcine precision-cut lung slice model (PCLS). Both models mimic the *in vivo* respiratory epithelium very closely, as they consist of ciliated and mucus-producing respiratory epithelial cells. The main advantage of ALI cultures is the formation of a barrier and the possibility to assess its integrity by resistance measurements or immunofluorescence microscopy (16). The benefit of PCLS is the preservation of the original tissue architecture and functionality, the presence of resident immune cells and other cell types, as well as the possibility to monitor the activity of ciliated cells by light microscopy (17).

Thus, we made use of the advantages of both models and found that DNT is neither involved in the reduction of the ciliary activity nor in the destruction of the respiratory epithelial barrier during *B. bronchiseptica* mono-infection. Moreover, DNT does not contribute to colonization or to immune activation of the respiratory epithelium by *B. bronchiseptica*. Consequently, we found no evidence that DNT of *B. bronchiseptica* facilitates co-infection with *S. suis*. Notably, activation of pro-inflammatory cytokines was clearly induced after infection with *B. bronchiseptica,* but not *S. suis,* whereas higher levels of some cytokines (e.g., IL-1α, CXCL8) were detected after co-infection with both pathogens.

## MATERIALS AND METHODS

### Bacterial strains

The wild-type strain *B. bronchiseptica* KM22 (WT) is a virulent phase I isolate from a swine herd with clinical atrophic rhinitis. The *dnt*-deficient mutant strain KM24 (Δ*dnt*; in a previous study designated as “KB24”) was generated by triparental mating and insertion of a 3.7-kb Gen^r^/oriT cassette (11). Both strains were kindly provided by Susan L. Brockmeier (National Animal Disease Center, USA). Illumina next-generation sequencing was performed to confirm the disruption of the *dnt* gene in the mutant strain KM24. Raw sequence data have been deposited in the NCBI Sequence Read Archive under accession number PRJNA1214268, and the genome of KM24 is available in NCBI GenBank under accession number CP181209. Both strains were grown on Columbia agar plates supplemented with 7% sheep blood (Oxoid, Thermo Fisher Scientific, Cat. No. PB5008A) for 48 h at 37°C under aerobic conditions as previously described (8). For infection experiments, cryo-preserved bacteria were used, and infection stocks were prepared as previously described (8), with minor modifications. Briefly, *B. bronchiseptica* WT and Δ*dnt* were grown overnight at 37°C on a horizontal shaker at 150 rounds per minute (rpm) under aerobic conditions in nutrient broth (NB; see Table S1 at https://doi.org/10.5281/zenodo.18400453), adjusted to an optical density of 0.05 at 600 nm (OD_600_) in pre-warmed NB and further incubated at 37°C and 150 rpm until OD_600_ of 0.5–0.6. Then, the bacterial culture was centrifuged (5,000 × *g*), and the pellet was re-suspended in NB with 15% (vol/vol) glycerol (Carl Roth, Cat. No. 3783.2). Aliquots were shock-frozen in liquid nitrogen and stored at −80°C.

For co-infection experiments, we used the virulent *S. suis* serotype 2 wild-type strain 10 (*S. suis* 10), which was kindly provided by Hilde Smith (formerly Wageningen University and Research, The Netherlands). *S. suis* was grown on Columbia agar plates supplemented with 7% sheep blood (Oxoid, Thermo Fisher Scientific, Cat. No. PB5008A) overnight at 37°C under aerobic conditions, and cryo-preserved bacterial stocks were prepared at the late exponential growth phase (OD_600_ of 1.0) as previously described (10).

### Preparation and infection of air-liquid interface (ALI) cultures

Primary porcine bronchial (PBEC) and tracheal epithelial cells (PTEC) were isolated from freshly slaughtered swine lungs according to Meng et al. (18) and as previously described (19), with some modifications. Lungs were obtained from apparently healthy pigs from a local slaughterhouse (Leine-Fleisch GmbH, Laatzen, Germany). Segments of the main bronchi or trachea were freed from tissue residues and digested in incubation medium (see Table S1 at https://doi.org/10.5281/zenodo.18400453) for 48 h at 4°C. PTEC/PBEC were harvested by scraping the cells from the luminal surface using a scalpel blade and cultivated in a T75 collagen I (Merck, Cat. No. C3867)-coated cell culture flask in Airway Epithelial Cell Basal Medium (AEBM; PromoCell, Cat. No. C21260) supplemented with several growth factors and antibiotics (Airway Epithelial Cell Growth Medium, AEGM; [see Table S1 at https://doi.org/10.5281/zenodo.18400453]) at 37°C and 5% CO_2_ in a humidified atmosphere. When cells reached confluence after approximately 5 days, they were detached using 0.05% trypsin-EDTA (TE; stock 0.5%, Thermo Fisher Scientific, Cat. No. 15400054) and were either used for ALI cultures or cryo-preserved in AEGM supplemented with 40% fetal calf serum (FCS; Biochrom, Cat. No. S 0615) and 10% dimethyl sulfoxide (DMSO; Merck, Cat. No. D2650) and stored in liquid nitrogen. For ALI cultures, cells were thawed, cultivated in AEGM until confluence, and then dissociated using 0.05% TE (Thermo Fisher Scientific, Cat. No. 15400054). Subsequently, 2.5 × 10^5^ cells were seeded on cell culture inserts with collagen IV (Merck, Cat. No. C7521)-coated polycarbonate membranes (6.5 mm diameter, 0.4 μm pore size; VWR, Cat. No. 734–2742) and incubated under submerged conditions in AEGM at 37°C and 5% CO_2_ for 4–5 days. Then, cells were introduced to ALI conditions by adding ALI medium (see Table S1 at https://doi.org/10.5281/zenodo.18400453) to the basal compartment only. Under these conditions, cells were maintained for another 3–4 weeks at 37°C and 5% CO_2_ to differentiate into a pseudostratified epithelium containing mucus-producing and ciliated cells. Meanwhile, ALI medium in the basal compartment was changed every 2–3 days, and cells were washed once a week with Hank’s Balanced Salt Solution (Thermo Fisher Scientific, Cat. No. 14025100) to remove excessive mucus and dead cells. Epithelial barrier integrity was assessed by measurement of the transepithelial electrical resistance (TEER) using a Millicell ERS-2 voltohmmeter (Merck Millipore).

**Fig 1:**
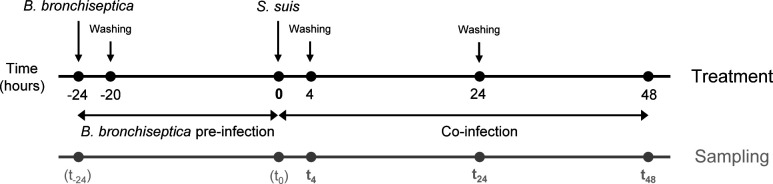
Schematic timeline of treatment and sampling during experimental infection of ALI cultures and PCLS with *B. bronchiseptica* and *S. suis*.

Prior to infection experiments, the differentiated ALI cultures were washed and maintained for at least 1 day without antibiotics. For co-infection experiments, ALI cultures were first infected apically with 10^3^ CFU/filter of *B. bronchiseptica* WT or Δ*dnt*, respectively, for 4 h. Then, non-adherent bacteria were washed away, and the cells were incubated further for 20 h under ALI conditions at 37°C and 5% CO_2_. Previous experiments have shown that this infection dose is sufficient to colonize the system but does not damage the epithelial cells substantially. Moreover, it has been shown in a porcine precision-cut lung slice (PCLS) model that a period of 4 h is sufficient for bacterial adherence and that *B. bronchiseptica* can colonize the respiratory epithelium within 24 h, meanwhile reducing the ciliary activity (8). The next day, ALI cultures were co-infected with 10^7^ CFU/filter of *S. suis* 10 for 4 h, corresponding to previous studies performed with this strain (9, 18). Afterward, cells were washed to remove non-adherent *Streptococci* and incubated further for up to 48 h under ALI conditions at 37°C and 5% CO_2_. After 48 h of co-infection, bacterial growth and colonization reached their maximum, and the epithelial barrier was broken down, which made it necessary to stop the experiment. Samples were taken after 4 h (t_4_), 24 h (t_24_), and 48 h (t_48_) of co-infection (Fig. 1).

### Preparation and infection of PCLS

PCLS were prepared from lungs obtained from apparently healthy pigs from a local slaughterhouse (Leine-Fleisch GmbH) as previously described (8).

Ciliary activity was monitored by light microscopy (Leica DMi1; Leica), and slices with at least 80% ciliary activity were chosen for infection experiments. Prior to infection, slices were kept in Roswell Park Memorial Institute (RPMI 1640) medium (Thermo Fisher Scientific, Cat. No. 21,875,034) without antibiotics for at least 1 day. Infection of PCLS was performed in accordance with the infection of ALI cultures described above (Fig. 1). In line with our previous study (8), the infection dose of *B. bronchiseptica* WT and Δ*dnt*, respectively, was 10^4^ CFU/slice, and for *S. suis* 10^7^ CFU/slice.

### Bacterial growth and colonization of *S. suis* and *B. bronchiseptica*

To distinguish colonies from *S. suis* and *B. bronchiseptica*, we used Columbia agar plates supplemented with 7% sheep blood and Oxoid Staph/Strep selective supplement (Thermo Fisher Scientific, Cat. No. SR0070E; referred to as “Strep-agar plate”) for *S. suis* as previously described (8). For *B. bronchiseptica*, we chose Columbia agar plates supplemented with 5% bovine blood, 100 mg/L bacitracin, 1 mg/L lincomycin, and 1 mg/L crystal violet (referred to as “Bordetella-agar plate”). All agar plates were incubated at 37°C under aerobic conditions for 48 h.

In order to quantify cell-associated (adherent and/or intracellular) bacteria in ALI cultures, cells were washed, detached by using 0.05% TE (Thermo Fisher Scientific, Cat. No. 15,400,054), and then lysed by adding 1% saponin (Carl Roth, Cat. No. 4185.1) and rigorous pipetting up and down as previously described (19). Cell lysates were serially diluted and plated on Strep- or Bordetella-agar plates, respectively.

To quantify tissue-associated bacteria (surface-adherent and/or intracellular bacteria) in infected PCLS, the slices were washed once and then homogenized in phosphate-buffered saline (PBS; Thermo Fisher Scientific, Cat. No. 14,190,250) using Lysing Matrix D (MP Biomedicals, Cat. No. 1169130-CF) and the FastPrep-24 5 G Instrument (3 × 30 s, 4.5 m/s; MP Biomedicals) as previously described (8). Afterward, bacterial number in the lysate was determined by serial dilution and replicate plating on Strep- and Bordetella-agar plates, respectively.

Similarly, supernatants of infected PCLS or ALI cultures were serially diluted and plated on agar plates to determine the number of CFU in the supernatant. To collect the supernatant from ALI cultures, ALI medium was added to the apical compartment, and ALI cultures were incubated on a horizontal shaker for 5 min at room temperature (RT).

### Cytotoxicity assay

To determine the cytotoxic effects caused by *B. bronchiseptica* and/*or S. suis*, we measured the release of lactate dehydrogenase (LDH) using the CytoTox 96 Non-Radioactive Cytotoxicity Assay (Promega, Cat. No. G1780) as previously described (8). LDH release of infected PCLS/ALI cultures was normalized to uninfected control samples, and the results were expressed as percentage LDH release compared to uninfected samples lysed with 1% (vol/vol, ALI cultures) or 10% (vol/vol, PCLS) Triton X 100 (Carl Roth, Cat. No. 3051.3) in medium, respectively. OD values were measured at 492 nm using SpectraMax i3x (Molecular Devices).

### Immunofluorescence analysis

Prior to whole mount immunofluorescence staining, PTEC/PBEC were washed and fixed with 3.7% (vol/vol) formaldehyde (stock solution 37%; Carl Roth, Cat. No. CP10.1). Then, ALI cultures were incubated for 1–2 h in blocking buffer (see Table S2 at https://doi.org/10.5281/zenodo.18400453) at RT. Primary and secondary antibodies (see Table S2 at https://doi.org/10.5281/zenodo.18400453) were diluted in antibody dilution buffer (see Table S2 at https://doi.org/10.5281/zenodo.18400453), added to the apical compartment, and incubated overnight at 4°C. Nuclei were stained with 4′,6-diamidino-2-phenylindole (DAPI, 0.5 μg/mL; Cell Signaling Technology, Cat. No. 4083). Finally, the membrane was cut out and mounted with ProLong Gold Antifade Reagent (Cell Signaling Technology, Cat. No. 9071), as previously described (19).

PCLS were fixed with 4% (vol/vol) phosphate-buffered formalin (Department of Pathology, University of Veterinary Medicine Hannover, Germany), embedded in paraffin (Engelbrecht, Cat. No. 17932a), and sections of 3–4 µm were prepared. Rehydration of paraffin sections, antigen retrieval, and immunofluorescence staining were performed as previously described (8).

Buffers and antibodies used to visualize cilia and bacteria are listed in Table S2 at https://doi.org/10.5281/zenodo.18400453.

Samples were analyzed using the Keyence BZ-X810 fluorescence microscope (Keyence) equipped with the Keyence Plan Apochromat 10×/0.45 or Plan Apochromat 40×/0.95 air objective lens. Image stacks with a z-distance of 0.3–0.4 µm were merged, and brightness, contrast, and colors were adjusted using BZ-X800 Analyzer software (version 1.1.2.4, Keyence). Optical sectioning (confocal-like technology) was applied for structural illumination and high-definition images of ALI cultures.

### Reverse-transcriptase quantitative real-time PCR (RT-qPCR)

The expression of cytokine genes and *dnt* was analyzed using quantitative real-time PCR following reverse transcription of RNA.

For total RNA extraction of ALI cultures, cells were lysed with TRI Reagent (Zymo Research, Cat. No. R2050-1-200) and RNA purification was performed using the Direct-zol RNA MiniPrep Kit (Zymo Research, Cat. No. R2052), including an on-column treatment with DNase I (Qiagen, Cat. No. 79254). RNA concentration and purity (260/280 and 260/230 ratios) were assessed by spectrophotometric analysis using SpectraMax i3x (Molecular Devices). Extracted RNA was stored at -80°C until further analysis.

RNA extraction from *B. bronchiseptica* cultures was carried out in the same way, with an additional lysing step using 0.5 g zirconium beads (Carl Roth, Cat. No. N037.1) and the FastPrep-24 5 G Instrument (2 × 30 s, 6 m/s; MP Biomedicals) prior to RNA purification.

Total RNA extraction from PCLS was performed according to the procedures described by Weldearegay et al. (20). PCLS were stored in RNA*later* (Merck, Cat. No. R0901) at -80°C until RNA extraction and washed with PBS prior to RNA extraction to remove any residues of RNA*later*. Slices were first homogenized in RLT buffer (Qiagen; Cat. No. 79,216) using Lysing Matrix D (MP Biomedicals, Cat. No. 1169130-CF) and the FastPrep-24 5 G Instrument (2 × 40 s, 6.5 m/s; MP Biomedicals). One volume of phenol/chloroform/isoamyl alcohol (Carl Roth, Cat. No. A156.1) was added to the lysate, mixed by shaking, and centrifuged at 12,000 × *g* for 5 min at RT. The aqueous phase was carefully transferred to a new tube, and one volume of chloroform/isoamyl alcohol (49:1; Carl Roth, Cat. No. 6340.1 and Cat. No. T870.1) was added, mixed by shaking, and centrifuged at 12,000 × *g* for 5 min at RT. The aqueous phase was carefully transferred to a new tube, and purification of RNA was continued using the MagMAX96 for Microarrays Total RNA Isolation Kit (Thermo Fisher Scientific, Cat. No. AM1839) according to the manufacturer’s instructions. RNA concentration and purity (260/280 and 260/230 ratio) were assessed by spectrophotometric analysis using SpectraMax i3x (Molecular Devices), and extracted RNA was stored at −80°C until further analysis.

The cDNA was prepared from a total of 500 ng RNA for cytokines and 1 µg for *dnt*.

For transcription of RNA from ALI cultures and bacterial cultures, we used 200 units M-MLV (H-) Point Mutant reverse transcriptase (Promega, Cat. No. M3682), dNTP Mix (10 mM each; Carl Roth, Cat. No. K039.1) and 500 ng Random Primers (Promega, Cat. No. C1181). For no-RT controls, the reverse transcriptase was replaced by RNase-free water. NoRT- and cDNA samples were diluted 1:4 in DNase-free water and stored at −20°C.

RNA samples from PCLS were transcribed using the QuantiTect Reverse Transcription Kit (Qiagen, Cat. No. 205311) according to the manufacturer’s instructions, including DNA digestion with the gDNA Wipeout Buffer provided by the kit. For no-RT controls, the Quantiscript reverse transcriptase was replaced by RNase-free water. NoRT- and cDNA samples were diluted 1:10 in DNase-free water and stored at −20°C.

Quantitative real-time PCR was performed in 20 µL reaction volumes containing 5 µL of diluted cDNA/noRT-sample, 500 nM gene-specific primers (see Table S3 at https://doi.org/10.5281/zenodo.18400453), and 10 µL of QuantiTect SYBR Green PCR Master Mix (Qiagen, Cat. No. 204245) with the Mx3005P Real-Time PCR System (Agilent Technologies). The PCR program consisted of an initial enzyme activation step at 95°C for 20 min, followed by 40 cycles of 30 s denaturation at 95°C, 30 s annealing at 55°C, and 30 s elongation at 72°C, and a melting curve for 1 min at 95°C, 30 s at 55°C, and 30 s at 95°C. All samples were run in duplicates, and non-template as well as noRT-controls were included. Relative gene expression of the cytokines was calculated by normalization to *GAPDH* transcript levels and to uninfected control samples (ΔΔC_q_) (21), and relative gene expression of *dnt* was calculated by normalization to the reference gene *adk* (ΔC_q_).

### Reverse-transcriptase PCR (RT-PCR)

Expression of the T-type voltage-gated calcium channels Ca_V_3.1 (Gene ID: 100513431, *CACNA1G*) and Ca_V_3.2 (Gene ID: 110259950, *CACNA1H*), which are predicted receptors for the DNT of *B. pertussis* (3), was analyzed using PCR following reverse transcription of RNA. RNA isolation and cDNA transcription were performed as described above. Genomic DNA (gDNA) of ALI cultures and PCLS was isolated using the DNeasy Blood and Tissue Kit (Qiagen, Cat. No. 69506) according to the manufacturer’s instructions (*Spin-Column Protocol for Purification of Total DNA from Animal Blood or Cells*). cDNA and gDNA samples from PCLS or ALI cultures infected with *B. bronchiseptica* WT for 72 h (t_48_) were then amplified with 0.5 units of HotStarTaq *Plus* DNA Polymerase (Qiagen, Cat. No. 203605) and 200 nM of each primer (see Table S3 at https://doi.org/10.5281/zenodo.18400453) using the Peqlab Advanced Primus 96 cycler (PEQLAB Biotechnologie GmbH, today part of VWR) as follows: initial enzyme activation step at 95°C for 15 min, followed by 45 cycles of 30 s denaturation at 94°C, 30 s annealing at 55°C, and 1 min elongation at 72°C for *CACNA1G* and annealing at 68°C, and 40 s elongation at 72°C for *CACNA1H*. Non-template and no-RT controls were included, as well as gDNA from uninfected ALI cultures and PCLS as a positive control. PCR products were loaded on a 2% agarose gel stained with ROTI GelStain (Carl Roth, Cat. No. 3865.2) and visualized on the Intas Gelstick imager (Intas).

### Enzyme-linked immunosorbent assay (ELISA)

The level of the cytokines IL-1α, IL-6, CXCL8, and TNF-α in the supernatant of infected PCLS was measured using porcine DuoSet ELISA kits (R&D Systems, Cat. No. DY680, DY686, DY535, and DY690B) according to the manufacturer’s instructions. Samples were diluted 1:2, 1:10, or 1:20. The Substrate Reagent Pack (R&D Systems, Cat. No. DY999) was used to identify streptavidin-horseradish peroxidase coupled to detection antibodies, and the reaction was stopped after 20 min with 2 N H_2_SO_4_ (Carl Roth, Cat. No. X873.1). OD values were measured at 450 nm using SpectraMax i3x (Molecular Devices) and normalized to OD values at 540 nm. The concentration of cytokines (pg/mL) was calculated by interpolation of the normalized OD values to a standard curve using the four-parameter logistic (4-PL) curve-fit model and the software GraphPad Prism version 10.4.1 for Windows (GraphPad Software).

The amount of supernatant from infected ALI cultures was not sufficient to perform ELISA.

### Statistical analysis

Data are shown as medians from 3 to 4 independent experiments (in technical duplicates). All statistical analyses were carried out using GraphPad Prism version 10.4.1 for Windows (GraphPad Software). Statistical significance was analyzed using the Mann-Whitney test or Kruskal-Wallis test followed by Dunn’s multiple comparison test with a confidence level of 0.05. Exact *P* values are listed in Tables S4 and S5 at https://doi.org/10.5281/zenodo.18400453.

## RESULTS

### Expression of *dnt* in *Bordetella bronchiseptica* and expression of DNT receptors in the porcine respiratory tract

**Fig 2:**
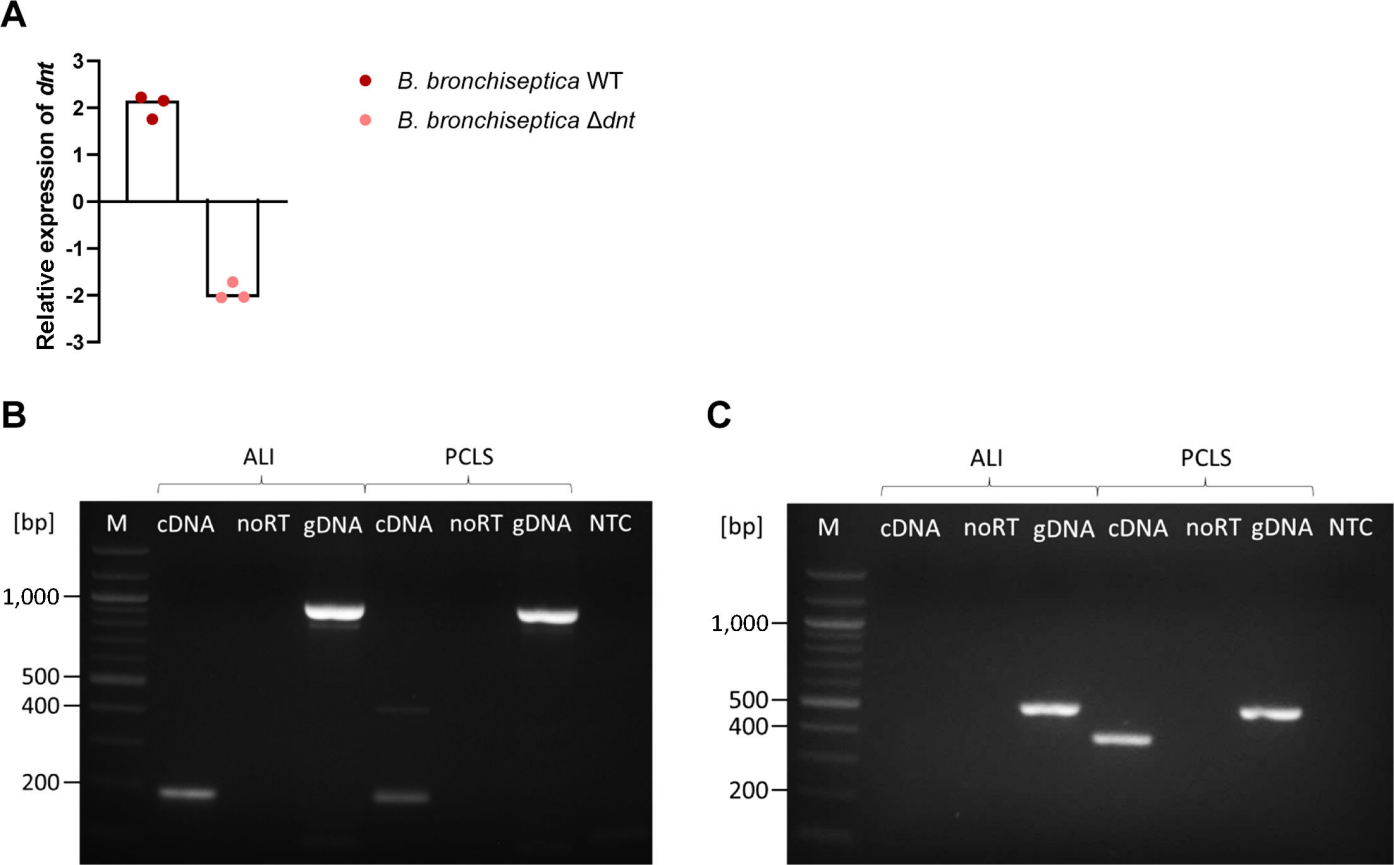
Expression of *dnt* during bacterial growth and expression of DNT receptors in ALI cultures and PCLS. (**A**) Expression of *dnt* during growth in nutrient broth was normalized to the expression of *adk*. The median of three independent experiments is shown. RT-PCR detection of (**B**) *CACNA1G* and (**C**) *CACNA1H* expression in ALI cultures and PCLS. M, marker; cDNA, standard RT-PCR reaction using reverse-transcribed RNA; noRT, negative control in which no reverse transcriptase was added to the RT reaction; gDNA, positive control in which genomic DNA was used as a template in the RT-PCR; NTC, non-template control.

To confirm the absence of dermonecrotic toxin (DNT) production in *B. bronchiseptica* Δ*dnt*, we analyzed the expression of *dnt* in both *Bordetella* strains grown to the stationary phase in nutrient broth via RT-qPCR, as DNT production has previously been shown in bacterial culture (22). In comparison to the reference gene *adk*, *dnt* was induced 2-fold in the *B. bronchiseptica* WT, whereas it was reduced 2-fold in the DNT-deficient mutant strain (Fig. 2A). Next, we checked whether the calcium channels Ca_V_3.1 (*CACNA1G*) and Ca_V_3.2 (*CACNA1H*), which are predicted receptors for the DNT of *B. pertussis* (3), are expressed in the herein used *in vitro* models—the porcine respiratory epithelial cells differentiated under air-liquid interface conditions (ALI cultures) and the porcine PCLS. For this, we isolated RNA from ALI cultures and PCLS and performed RT-PCR. The results demonstrated that *CACNA1G* is expressed in both models (Fig. 2B). On the contrary, *CACNA1H* is indeed present in the genomic DNA of both ALI cultures and PCLS but only expressed in PCLS (Fig. 2C). Taken together, in both models, at least one of the receptors needed for DNT binding is expressed, showing that these models are suitable to investigate the role of DNT during infection.

### Colonization of the porcine respiratory epithelium by *B. bronchiseptica* and *S. suis*

Since we have proven the presence of at least one of the predicted DNT-binding receptors in our *in vitro* models mimicking the porcine respiratory epithelium, we proceeded to study the interactions between *B. bronchiseptica* and *S. suis* as well as the role of DNT in a co-infection scenario. For this, ALI cultures as well as PCLS were initially infected with the virulent *B. bronchiseptica* wild-type strain (WT), which is positive for DNT, and the isogenic DNT-deficient mutant strain (Δ*dnt*), respectively, for 24 h. Subsequently, ALI cultures and PCLS were infected with the virulent *S. suis* serotype 2 wild-type strain 10 (*S. suis* 10).

**Fig 3:**
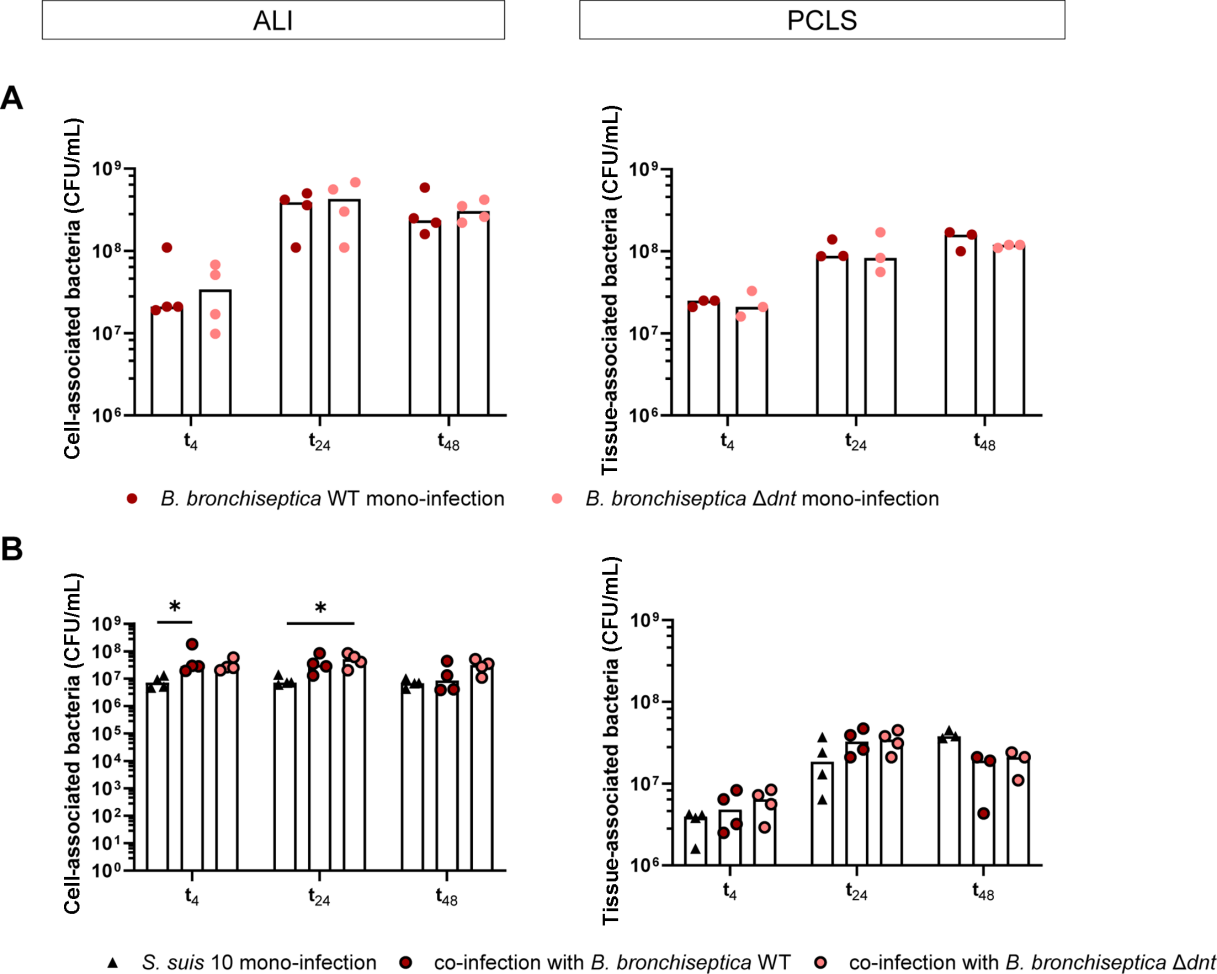
Association of *B. bronchiseptica* and *S. suis* to mono- and co-infected ALI cultures and PCLS. ALI cultures and PCLS were pre-infected with *B. bronchiseptica* WT and *B. bronchiseptica* Δ*dnt*, respectively, for 24 h. Subsequently, ALI cultures and PCLS were infected with *S. suis* strain 10 for up to 48 h. To calculate the number of cell-/tissue-associated bacteria, cell/tissue lysates were plated on agar plates to determine CFU/mL of (**A**) *B. bronchiseptica* in mono-infected samples and (**B**) *S. suis* in mono- and co-infected samples. The median of 3–4 experiments is shown. Significant differences between WT and Δ*dnt* as well as between *S. suis* mono-infection and co-infection were analyzed with (**A**) Mann-Whitney test or (**B**) Kruskal-Wallis test followed by Dunn‘s multiple comparisons test (* *P* < 0.05).

Plating of whole cell/tissue lysates revealed a time-dependent increase of cell- and tissue-associated *Bordetellae* in ALI cultures as well as in PCLS with a maximum of approximately 4 × 10^8^ CFU/mL in ALI cultures and approximately 1 × 10^8^ CFU/mL in PCLS after 48 h of infection (t_24_; Fig. 3A). The number of cell-/tissue-associated *Bordetellae* was comparable for the wild-type strain and the mutant (Fig. 3A) but was slightly lower when ALI cultures or PCLS were co-infected with *S. suis* (see Fig. S1 at https://doi.org/10.5281/zenodo.18400453). In contrast, the number of *Bordetellae* in the supernatant of infected ALI cultures or PCLS was slightly increased when they were co-infected with *S. suis* (see Fig. S2B and C at https://doi.org/10.5281/zenodo.18400453), indicating that both bacterial species compete for binding sites and *S. suis* could displace *B. bronchiseptica* to some extent.

The number of cell-associated *Streptococci* remained almost similar in ALI cultures during the whole infection experiment (approximately 7 × 10^6^ CFU/mL) but was significantly increased when cells were pre-infected with *B. bronchiseptica* WT or Δ*dnt*, respectively (approximately 2 × 10^7^ CFU/mL; Fig. 3B). Colonization of PCLS by *S. suis* was time-dependent and enhanced by pre-infection with *B. bronchiseptica* WT or Δ*dnt* at t_4_ and t_24_ (Fig. 3B). Interestingly, at t_48_, the number of tissue-associated *Streptococci* was lower when PCLS were pre-infected with *B. bronchiseptica* WT or Δ*dnt* (Fig. 3B). In contrast, the number of *Streptococci* in the supernatant was significantly increased at t_24_ and t_48_ when PCLS were pre-infected with *B. bronchiseptica* WT or Δ*dnt* (see Fig. S2A at https://doi.org/10.5281/zenodo.18400453). In ALI cultures, the number of *Streptococci* in the supernatant was highest at t_4_ (approximately 1 × 10^9^ CFU/mL) and similar in the supernatant of mono- and co-infected cells. At later time points (t_24_ and t_48_), approximately 4 × 10^8^ CFU/mL of *S. suis* 10 were counted when ALI cultures were pre-infected with *B. bronchiseptica* WT or Δ*dnt*, respectively, but only 1 × 10^8^ CFU/mL in the supernatant of mono-infected ALI cultures (see Fig. S2A at https://doi.org/10.5281/zenodo.18400453).

**Fig 4:**
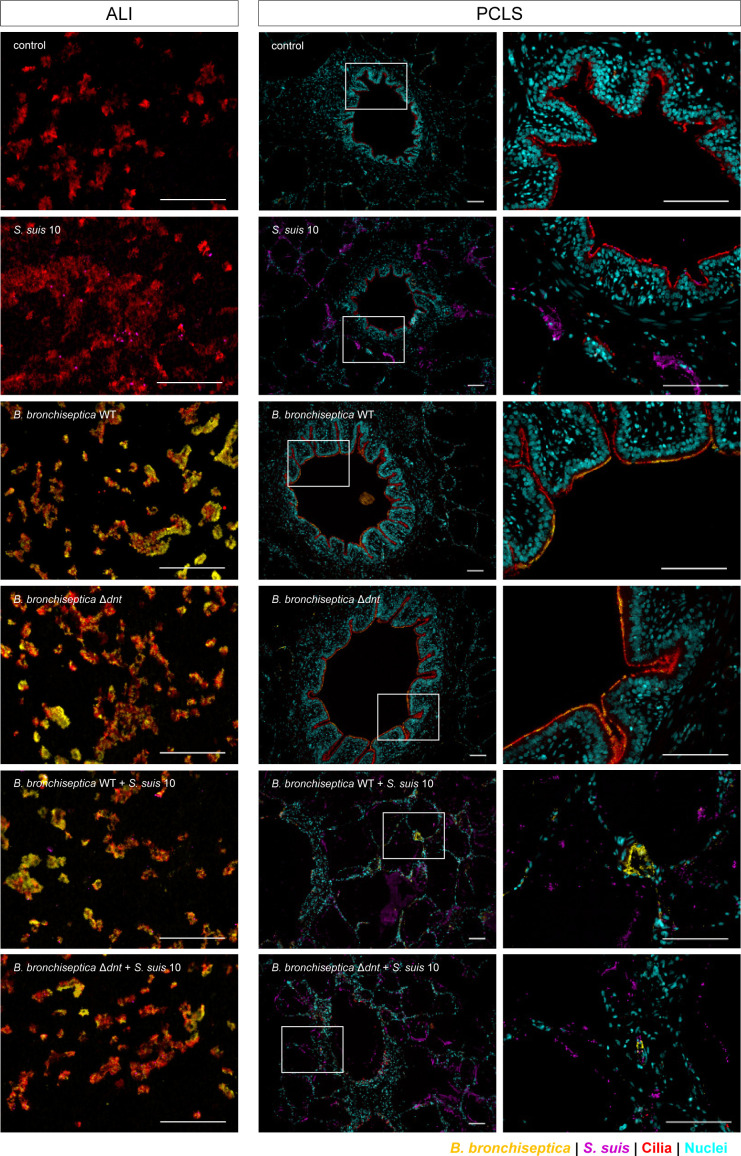
Visualization of bacterial association to mono- and co-infected ALI cultures and PCLS. Immunofluorescence staining of ALI cultures at t_4_ (4 h post-co-infection) and PCLS at t_24_. *Bordetellae* are shown in yellow, *Streptococci* in purple, cilia (α-tubulin) in red, and nuclei (DAPI) in cyan; bars represent 100 µm. Boxed regions in PCLS images are depicted with a higher magnification in the image to the right.

Immunofluorescence analysis confirmed these plating results (Fig. 4). Moreover, visualization of the bacteria revealed that *B. bronchiseptica* WT and Δ*dnt* both adhered to the cilia in masses in mono-infected PCLS, whereas when co-infected with *S. suis* for 24 h, *Bordetellae* as well as ciliated cells could hardly be detected. On one hand, we assume that cilia are destroyed during co-infection, and as a result, the preferred structures to bind to are missing for *B. bronchiseptica*. On the other hand, we cannot exclude technical issues during the process of immunofluorescence staining (e.g., loss of bacteria during washing steps). *Streptococci* were mainly found in the alveolar epithelium of PCLS (Fig. 4). In ALI cultures, *B. bronchiseptica* preferentially adhered to cilia, as the WT and the mutant strains were almost exclusively found on top of them (Fig. 4). After prolonged infection, almost all cilia were destroyed by *B. bronchiseptica*, independent of the presence of DNT (see Fig. S3 at https://doi.org/10.5281/zenodo.18400453). Notably, immunofluorescence analysis of ALI cultures did not confirm the enhancing effect of *B. bronchiseptica* pre-infection on the colonization capacity of *S. suis* that we found by plating of cell lysates. As mentioned above, this could be due to the immunofluorescence staining procedure or due to difficulties in detecting *Streptococci* via fluorescence microscopy. Thus, the determination of cell-associated bacteria via plating of cell lysates seems to be the more reliable method.

In general, these results show that *B. bronchiseptica* colonizes the porcine respiratory epithelium *in vitro* more efficiently compared to *S. suis* and that DNT has no influence on the colonization capacity of *B. bronchiseptica*. However, pre-infection with *B. bronchiseptica* significantly promoted colonization capacity of *S. suis*, independent of the presence of DNT.

### Cytotoxic effects of *B. bronchiseptica* and *S. suis* on the porcine respiratory epithelium

Cytotoxic effects on respiratory epithelial cells upon infection with *B. bronchiseptica* and/or *S. suis* were determined by measuring the release of lactate dehydrogenase (LDH) into the supernatant of infected cells.

**Fig 5:**
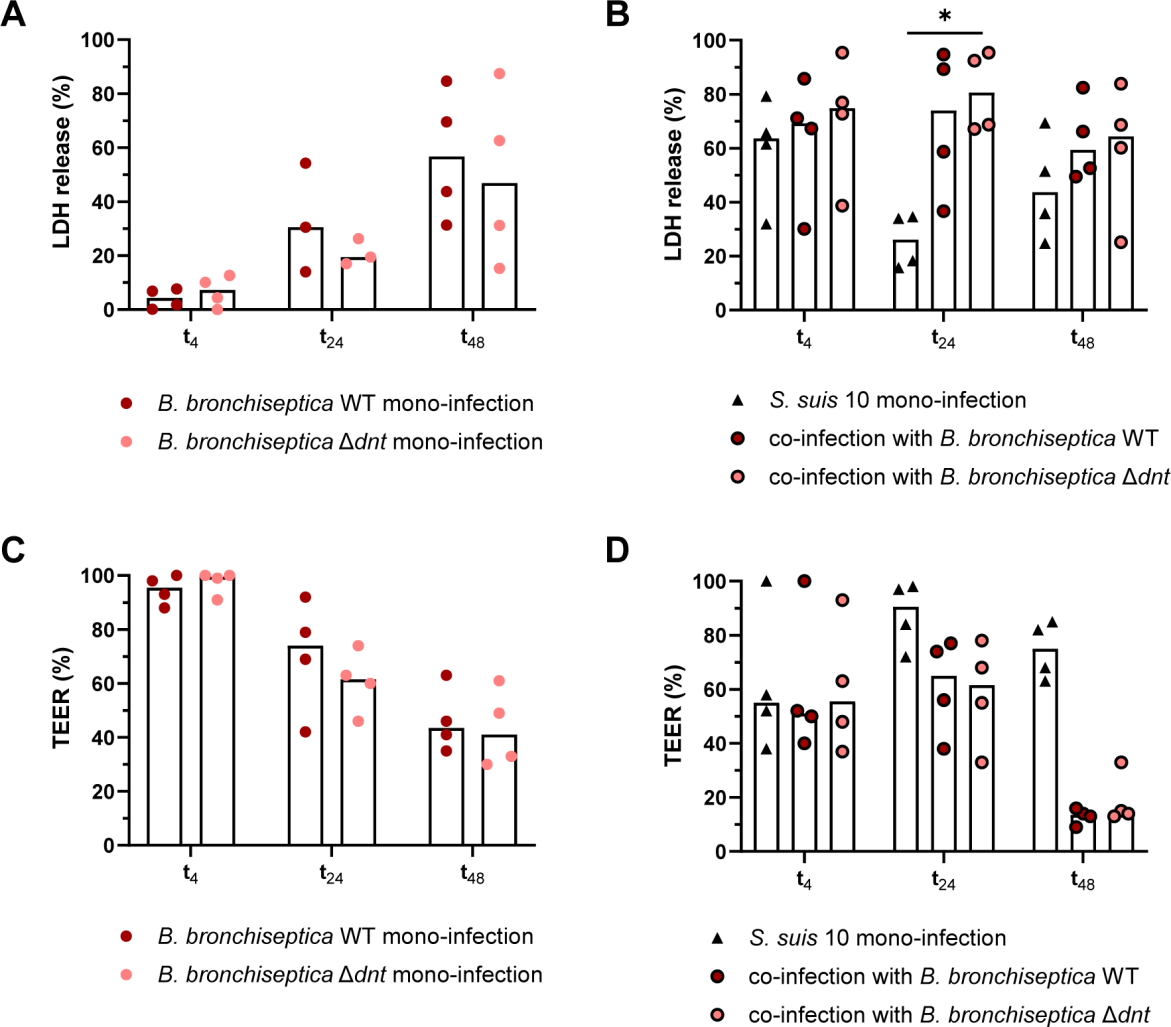
Cytotoxic effects of *B. bronchiseptica* and/or *S. suis* and expression of *dnt* during mono- and co-infection of ALI cultures. ALI cultures were pre-infected with *B. bronchiseptica* WT and *B. bronchiseptica* Δ*dnt*, respectively, for 24 h. Subsequently, ALI cultures were infected with *S. suis* strain 10 for up to 48 h. (**A and B**) Cytotoxic effects were determined by measuring the release of LDH into the supernatant of infected cells at the indicated times. The results are expressed as percentage LDH release compared to cells lysed with 1% Triton X-100 and normalized to non-infected cells. (**C and D**) Barrier integrity was evaluated by measuring the trans-epithelial electrical resistance (TEER) at the indicated times. The results are expressed as percentage TEER, normalized to non-infected cells. The median of 3–4 experiments is shown. Significant differences between WT and Δ*dnt* as well as between *S. suis* mono-infection and co-infection were analyzed with (**A and C**) Mann-Whitney test or (**B and D**) Kruskal-Wallis test followed by Dunn’s multiple comparisons test (* *P* < 0.05).

In ALI cultures, the amount of LDH released into the supernatant upon infection with *B. bronchiseptica* increased over time up to approximately 50% (normalized to cells lysed with 1% Triton X 100) after 72 h of infection (t_48_; Fig. 5A) but was similar for the WT and the mutant strain. The highest amount of LDH released upon infection with *S. suis* was detected after 4 h of infection (approximately 60%, Fig. 5B), corresponding to the high number of *Streptococci* in the supernatant at t_4_ (see Fig. S2A at https://doi.org/10.5281/zenodo.18400453). Cytotoxicity at t_4_ was slightly enhanced when cells were pre-infected with *B. bronchiseptica* WT or Δ*dnt*, respectively, although the difference was not significant (Fig. 5B). At t_24_, mono-infection with *S. suis* induced only approximately 20% LDH release, whereas co-infection with both pathogens resulted in approximately 75% LDH release (Fig. 5B), indicating a synergistic effect of the cytotoxicity induced by both pathogens. At t_48_, the difference between mono- and co-infected ALI cultures was not as obvious (approximately 40% LDH release in mono-infected and 60% in co-infected ALI cultures; Fig. 5B).

In addition to the LDH release assay, we defined the detrimental effects of *B. bronchiseptica* and/or *S. suis* on the epithelial barrier by measuring the trans-epithelial electrical resistance (TEER), a parameter for the barrier integrity, and compared it to non-infected ALI cultures (set as 100%). At t_4_, infection with *B. bronchiseptica* WT or Δ*dnt* alone had no effect on the barrier integrity (Fig. 5C), whereas infection with *S. suis* alone as well as co-infection with both pathogens resulted in a significant drop in the TEER values by approximately 50% (Fig. 5D). Interestingly, the barrier integrity recovered 20 h later to almost 100% when infected with *S. suis* alone. In contrast, infection with *B. bronchiseptica* WT or Δ*dnt* caused a time-dependent disturbance of the epithelial barrier (Fig. 5C), which was even more pronounced in cells co-infected with *S. suis*, resulting in TEER values as low as 10% at t_48_ (Fig. 5D).

**Fig 6:**
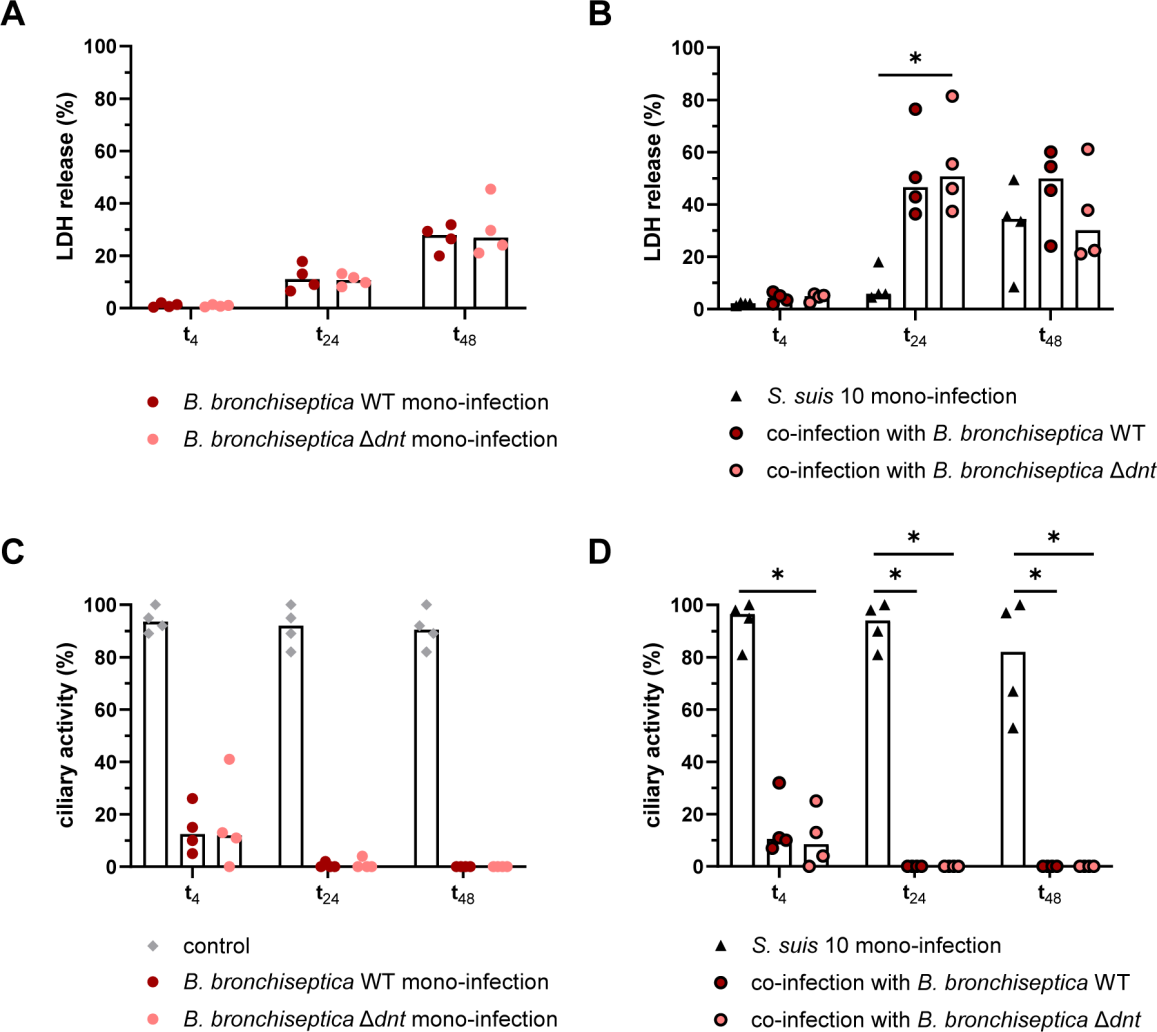
Cytotoxic effects of *B. bronchiseptica* and/or *S. suis* during mono- and co-infection of PCLS. PCLS were pre-infected with *B. bronchiseptica* WT and *B. bronchiseptica* Δ*dnt*, respectively, for 24 h. Subsequently, PCLS were infected with *S. suis* strain 10 for up to 48 h. (**A and B**) Cytotoxic effects were determined by measuring the release of LDH into the supernatant of infected slices at the indicated times. The results are expressed as percentage LDH release compared to PCLS lysed with 10% Triton X-100 and normalized to uninfected PCLS. (**C and D**) Ciliary activity of uninfected (control) and infected PCLS was monitored at the indicated times by estimating the ciliary beating using light microscopy. The results are expressed as percentage ciliary activity compared to the ciliary activity before infection (set as 100%). The median of 3–4 experiments is shown. Significant differences between WT and Δ*dnt* as well as between *S. suis* mono-infection and co-infection were analyzed with (**A and C**) Mann-Whitney test or (**B and D**) Kruskal-Wallis test, followed by Dunn’s multiple comparisons test (* *P* < 0.05).

In accordance with the infection of ALI cultures, we determined the cytotoxic effects of *B. bronchiseptica* and/or *S. suis* infection on PCLS by measuring the release of LDH into the supernatant of infected PCLS. Only low amounts of LDH were detected after infection with *B. bronchiseptica* WT or Δ*dnt*, approximately 10% after 48 h (t_24_) and approximately 25% after 72 h of infection (t_48_; Fig. 6A). Infection with *S. suis* alone resulted in similar percentages of LDH release, approximately 5% at t_24_ and approximately 35% at t_48_ (Fig. 6B). At t_4_, almost no LDH was measurable in the supernatants of PCLS infected with *B. bronchiseptica* or *S. suis* alone (Fig. 6B). However, co-infection with both pathogens led to synergistic cytotoxic effects, indicated by a significant increase of LDH release at t_4_ and t_24_ (Fig. 6B). At t_48_, the amount of LDH was lower in PCLS co-infected with *B. bronchiseptica* Δ*dnt* and *S. suis* compared to PCLS co-infected with *B. bronchiseptica* WT and *S. suis,* but in all other samples, no difference between the WT and the mutant strain was detectable (Fig. 6A and B).

One special feature of PCLS is the possibility to monitor ciliary beating by light microscopy, which provides information on the fitness and vitality of the tissue. We monitored the ciliary activity during infection and observed that both *B. bronchiseptica* strains had detrimental effects on the ciliary activity as it was reduced by approximately 60% after 24 h of infection (immediately before co-infection with *S. suis* (see Fig. S4 at https://doi.org/10.5281/zenodo.18400453) and by even 100% after 48 h of infection (t_24_; Fig. 6C). In contrast, infection with *S. suis* alone had almost no effect on the ciliary activity after 4 h and 24 h. Only after prolonged infection with *S. suis* (48 h), ciliary activity was reduced by approximately 30% (Fig. 6D).

Additional experiments showed that LDH is only released upon treatment of PCLS with whole bacterial culture of *B. bronchiseptica* WT but not with cell-free (heat-inactivated) supernatant (see Fig. S5 at https://doi.org/10.5281/zenodo.18400453). This indicates that cytotoxic effects of *B. bronchiseptica* were not induced by a secreted factor but are dependent on the presence of living bacterial cells. Accordingly, ciliary activity was only abolished when PCLS were treated with bacterial culture but not with cell-free (heat-inactivated) supernatant (see Fig. S5 at https://doi.org/10.5281/zenodo.18400453).

Taken together, *B. bronchiseptica* infection had harmful effects on the ciliary beating, which were independent of DNT and did not correspond to the cytotoxic effects, which were very low. However, co-infection with both pathogens led to significantly increased damage of the respiratory epithelium.

### Cytokine response of the porcine respiratory epithelium infected with *B. bronchiseptica* and/or *S. suis*

During the early stages of bacterial infection, the host usually reacts by expressing pro-inflammatory cytokines, such as *IL-1α*, *IL-6*, *CXCL8*, and *TNF-α*. Thus, we were also interested in studying the pro-inflammatory host cell response during co-infection with *B. bronchiseptica* and *S. suis* and whether it is affected by DNT. For this, we studied the induction of respective cytokine genes in ALI cultures as well as PCLS upon infection with *B. bronchiseptica* and/or *S. suis* by RT-qPCR.

**Fig 7:**
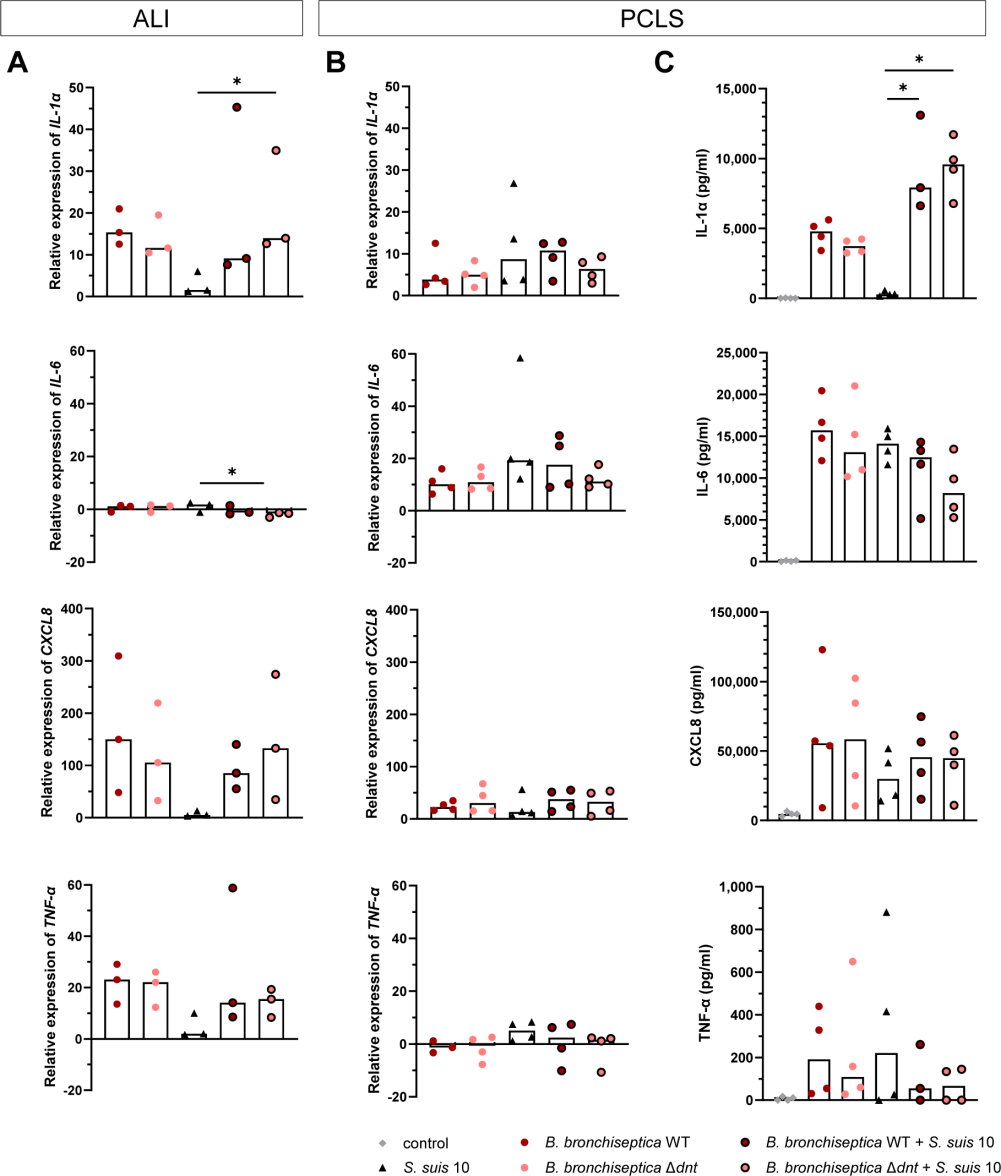
Pro-inflammatory cytokine response toward *B. bronchiseptica* and *S. suis* mono- and co-infection of ALI cultures and PCLS. (**A**) ALI cultures and (**B**) PCLS were pre-infected with *B. bronchiseptica* WT and *B. bronchiseptica* Δ*dnt*, respectively, for 24 h. Subsequently, ALI cultures and PCLS were infected with *S. suis* strain 10 for up to 48 h. Gene expression levels of *IL1α*, *IL-6*, *CXCL8*, and *TNF-α* were analyzed at t_24_ (24 h post co-infection) by RT-qPCR and normalized to the expression of *GAPDH,* and relative fold differences were calculated compared to non-infected cells. (**C**) Cytokine levels in the supernatant of uninfected (control) and infected PCLS were quantified at t_24_ using ELISA. The median of 3–4 independent experiments is shown. Significant differences between WT and Δ*dnt* as well as between *S. suis* mono-infection and co-infection were analyzed with the Kruskal-Wallis test, followed by Dunn’s multiple comparisons test (* *P* < 0.05).

In ALI cultures at t_4_, only minor changes in the expression of *IL-1α* and *TNF-α* were observed, but *CXCL8* was activated by both pathogens and highly expressed in co-infected epithelial cells (see Fig. S6A at https://doi.org/10.5281/zenodo.18400453). At t_24_, expression of *IL-1α*, *CXCL8*, and *TNFα* was mainly induced by *B. bronchiseptica* but barely by *S. suis* (Fig. 7A). At t_48_, we detected higher levels of *IL-1α*, *CXCL8*, and *TNF-α* in co-infected cells compared to cells infected with only one of the pathogens (see Fig. S7A at https://doi.org/10.5281/zenodo.18400453). Expression of *IL-6* was only induced by infection with *S. suis* after prolonged infection (Fig. 7A; see Fig. S7A at https://doi.org/10.5281/zenodo.18400453).

In contrast to ALI cultures, which consist of epithelial cells only, PCLS provides a composition of different cell types, including epithelial cells, endothelial cells, fibroblasts, as well as some resident immune cells (23, 24). Therefore, we assumed that the pro-inflammatory cytokine response upon infection of PCLS differs from the cytokine response in ALI cultures described above.

In PCLS at t_24_, the expression of *IL-1α*, *IL-6*, and *TNF-α* was mainly induced by infection with *S. suis* and, to a lesser extent, by infection with *B. bronchiseptica* alone. In contrast, *CXCL8* was mainly stimulated by infection with *B. bronchiseptica* and less after infection with *S. suis* alone (Fig. 7B). Expression of *IL-1α*, *IL-6*, and *CXCL8* was induced by both pathogens at t_4_, and all these cytokines were significantly higher expressed when slices were co-infected with *B. bronchiseptica* and *S. suis* compared to PCLS infected with *S. suis* alone. Expression of *TNF-α* was only slightly induced after mono- and co-infection with *S. suis* but not by infection with *B. bronchiseptica* alone (see Fig. S6B at https://doi.org/10.5281/zenodo.18400453). At t_48_, expression of all cytokines was only activated in PCLS infected with *S. suis* alone but not (or only to a lesser extent) in PCLS infected with *B. bronchiseptica* or slices that were co-infected with both pathogens. Especially, the expression levels of *TNF-α* were significantly lower in co-infected PCLS compared to *S. suis* mono-infected PCLS (see Fig. S7B at https://doi.org/10.5281/zenodo.18400453).

When comparing the expression of cytokines in ALI cultures and PCLS, *IL-6* was mainly expressed in the PCLS model, whereas the expression of *CXCL8* was primarily activated in ALI cultures. *IL-1α* was expressed during early infection in PCLS but to a greater extent in ALI cultures after prolonged infection. *TNF-α* was rarely activated in both models but significantly downregulated after prolonged co-infection of PCLS (Fig. 7A and B; see Fig. S7A and B at https://doi.org/10.5281/zenodo.18400453).

The level of pro-inflammatory cytokines released into the supernatant of infected PCLS was analyzed using enzyme-linked immunosorbent assay (ELISA). At t_24_, we found only low amounts of TNF-α in the supernatant of PCLS and no correlation to the infecting pathogen, which fits the low expression level of *TNF-α*. In accordance with the high expression of *CXCL8*, we found very high levels of CXCL8 in the supernatant of PCLS, especially when infected with *B. bronchiseptica*. IL-6 was found in high levels in the supernatant, regardless of the infecting pathogen, and IL-1α was mainly released into the supernatant when PCLS were infected with *B. bronchiseptica* and was higher in co-infected PCLS (Fig. 7C). At t_4_, the highest levels of cytokines were found in PCLS that were co-infected with both pathogens (see Fig. S6B at https://doi.org/10.5281/zenodo.18400453). At t_48_, the levels of IL-1α, IL-6, and TNF-α were highest in PCLS infected with *S. suis* alone, which is in accordance with the expression data of these cytokines. Notably, the level of CXCL8 was highest in PCLS infected with *B. bronchiseptica* alone, whereas it was significantly lower in the supernatant of PCLS infected with *S. suis* alone or co-infected with both pathogens (see Fig. S7C at https://doi.org/10.5281/zenodo.18400453).

Taken together, expression and secretion of pro-inflammatory cytokines in our *in vitro* models during early infection were mainly induced by *B. bronchiseptica* and during late infection, mainly by *S. suis*. Co-infection with both pathogens, depending on the time and the analyzed cytokine, had a limited amplifying effect on cytokine expression as well as secretion and, in some cases, even an inhibiting effect. According to our results described above, we did not observe any noteworthy differences between *B. bronchiseptica* WT and the mutant strain, indicating that DNT is not involved in the activation of the investigated cytokines upon *B. bronchiseptica* infection.

## DISCUSSION

In a previous study, we clearly demonstrated that *B. bronchiseptica* can pave the way for *S. suis* infection (8). However, we could not yet clarify which virulence factor(s) of *B. bronchiseptica* is/are involved in this process. Thus, in this study, we set out to investigate the role of the dermonecrotic toxin (DNT) of *B. bronchiseptica* in mono- and co-infections of the porcine respiratory tract. Furthermore, we included primary porcine respiratory epithelial cells differentiated under air-liquid interface conditions (ALI cultures) in addition to the porcine PCLS.

In comparison to PCLS, ALI cultures offer the opportunity to investigate the effects of an infection with respiratory pathogens on the three-dimensional respiratory epithelial barrier and the interactions between the respective pathogen and respiratory epithelial cells in more detail. By using ALI cultures as an additional *in vitro* model of the porcine respiratory tract, we could show that *B. bronchiseptica* is able to disrupt the respiratory epithelial barrier, reflected by a decrease of the trans-epithelial electrical resistance (TEER) and the loss of ciliated cells, which might facilitate *S. suis* colonization and invasion of the respiratory epithelium as it is described for swine influenza virus (SIV) (10). Our findings are supported by the study of Cao et al., who found that *B. bronchiseptica* can impair the integrity of the tracheal epithelial cell barrier by cleavage of the E-cadherin adherence junction protein mediated by the bacterial protease HtrA/DegQ (25). Other research groups identified the adenylate cyclase toxin (ACT) (26) or the tracheal cytotoxin (TCT) in combination with lipopolysaccharides (LPS) (27) as the responsible factors for TEER reduction. However, in our study, advanced destruction of the epithelial barrier cannot be correlated with increased *S. suis* colonization. On the contrary, *S. suis* adherence was even lower after prolonged infection when TEER measurement indicated a break-down of the epithelial barrier. Interestingly, especially in co-infected ALI cultures, the reduction of the epithelial barrier integrity corresponds to the high amounts of lactate dehydrogenase (LDH) in the supernatant of infected cells, reflecting the cytotoxic activity of both pathogens that results in damage of the epithelial cells. The observed cytotoxic effects are time-dependent but not necessarily linked to increasing bacterial numbers, suggesting that an accumulation of the cytotoxic factors is required to harm the epithelial cells to this extent.

In both model systems mimicking the porcine respiratory epithelium, we confirmed the major findings of our previous study: (i) *B. bronchiseptica* has detrimental effects on ciliated epithelial cells, (ii) *B. bronchiseptica* can promote adherence and colonization of *S. suis*, and (iii) *B. bronchiseptica* can thereby promote cytotoxic effects of *S. suis* (8). At this point, it should be noted that we used a different *B. bronchiseptica* strain (*B. bronchiseptica* KM22, isolated from a pig with atrophic rhinitis) compared to our previous study (*B. bronchiseptica* 1263/2/18, obtained from a swine herd with unspecific symptoms), indicating that these effects are strain independent. Our findings are in line with a recent publication of Hau et al., who described that pre-inoculation of pigs with *B. bronchiseptica* KM22 increased nasal colonization with *S. suis*, although the incidence of *S. suis* disease was not augmented (28). *B. bronchiseptica* KM22 efficiently reduced the ciliary activity in PCLS, as we described it for *B. bronchiseptica* 1263/2/18 (8). Notably, additional experiments showed that only bacterial culture, but not cell-free (heat-inactivated) supernatant, is able to reduce ciliary activity. These findings are in contradiction to the long-lasting hypothesis that reduction of the ciliary activity (and destruction of cilia) upon infection with *B. bronchiseptica* is mediated by TCT, a heat-stable toxin that is released into the supernatant (29). However, further investigations are needed to clarify these contradictory findings. Moreover, although there seems to be a strong correlation between ciliary reduction by *B. bronchiseptica* and increased adherence of *S. suis*, further studies, for example, with a strain unable to impair ciliary activity, are needed to really demonstrate the link between these observations.

In the present study, we focused on the role of DNT in mono- and co-infection of the porcine respiratory epithelium. This toxin can be associated with the destruction of nasal conchae observed in atrophic rhinitis of pigs by activation of the small GTP-binding protein Rho, which results in alterations of the cytoskeleton of osteoblasts (5). Moreover, Brockmeier et al. postulated that DNT might contribute to *B. bronchiseptica* colonization of the porcine respiratory tract as the mutant strain deficient for DNT (the same strain as used in the herein study) showed a slightly reduced colonization capacity *in vivo* (11). Although the effects of DNT on the cytoskeleton of osteoblasts and its involvement in nasal turbinate atrophy are well known, and it is also conceivable that DNT induces alterations in the cytoskeleton of epithelial cells, which might result in an increased bacterial adherence, we could not observe such effects in our *in vitro* infection models. The mutant strain *B. bronchiseptica* Δ*dnt* (*B. bronchiseptica* KM24/KB24) showed similar colonization capacities as the wild-type strain, and we did not detect any evidence for cytoskeletal alterations of the epithelial cells. The same was true for co-infection of ALI cultures and PCLS with *B. bronchiseptica* and *S. suis*—the wild-type and the mutant strain showed similar colonization capacities, and both strains promoted adherence and colonization of *S. suis* to the same extent. A comparable outcome was observed in an *in vivo* co-infection study using a DNT-deficient mutant strain (the same strain as used in the herein study), demonstrating that DNT is not essential for predisposing pigs to infection with toxigenic *Pasteurella multocida* (30). In addition, we found that the wild-type strain and the DNT-deficient mutant strain induced similar amounts of LDH release upon infection of ALI cultures and PCLS, respectively, with *B. bronchiseptica* alone or co-infection with *S. suis*. This suggests that DNT does not play a role in cytotoxicity of *B. bronchiseptica* in the herein tested *in vitro* systems. However, this pathogen possesses several other toxins that were described to contribute to its cytotoxic capacity—TCT, ACT, and LPS (26, 27, 31). Almost 30 years ago, van den Akker described that cytotoxic activity toward epithelial cells appears to be regulated by the two-component signal transduction system BvgAS, and the factor responsible for the cytotoxic effect is not secreted into the culture supernatant, the latter being in accordance with our findings (32). To confirm the validity of the mutant strain, we performed whole genome sequencing (sequence available in NCBI GenBank under accession number CP18120) and analyzed the expression of *dnt* during growth in bacterial culture medium. Whole genome sequencing confirmed that the *dnt* gene is interrupted by a gentamycin cassette, and RT-qPCR revealed the expression of *dnt* in the wild-type strain but not in the mutant strain. A few years ago, Teruya et al. published that the T-type voltage-gated calcium channels Ca_v_3.1 and Ca_v_3.2 serve as receptors for DNT of *Bordetella pertussis* (3), and another group confirmed the presence of these receptors in human lung epithelial cells (7). Since the DNT of *B. bronchiseptica* and *B. pertussis* are nearly identical (99.0% amino acid sequence identity) (2–4), we assumed that the DNT of *B. bronchiseptica* can bind to these receptors likewise and checked whether they are present in the porcine respiratory tract. Indeed, RT-PCR revealed the expression of *CACNA1G* (encoding for Ca_v_3.1) in both ALI cultures and PCLS, as well as the expression of *CACNA1H* (encoding for Ca_v_3.2) in PCLS. Nevertheless, the direct interaction of *B. bronchiseptica* DNT with these two receptors remains to be proven in our cell culture systems, and further studies with recombinant DNT are needed to clarify the role of DNT. However, even if a direct interaction of DNT with respiratory epithelial cells could be proven, our results strongly suggest that DNT does not have the same effects on respiratory epithelial cells, as it has been described for osteoblasts. Moreover, it has to be considered that DNT-induced nasal turbinate atrophy seen *in vivo* is a chronic process that takes several weeks (11), whereas we analyzed the effects of DNT on respiratory epithelial cells during early infection within a few days only.

Finally, we were also interested in investigating the cytokine response during the early stages of co-infection with *B. bronchiseptica* and *S. suis* and whether DNT has an effect on this host cell response. We hypothesized that *B. bronchiseptica* pre-infection might alter the host’s immune response in a way that facilitates *S. suis* infection. *Bordetella* has been shown to induce the secretion of pro-inflammatory cytokines (e.g., TNF-α, IL-6, IL-12) as well as the anti-inflammatory cytokine IL-10. In this regard, recognition of LPS by the Toll-like receptor 4 (TLR4) plays an important role (33, 34). Other virulence factors that might be involved in the induction/secretion of pro- and anti-inflammatory cytokines are the ACT (26, 35) and the TCT (27). Notably, to our knowledge, no studies on the role of DNT in modulation of the host’s immune response, and no investigations on the immune response in the porcine respiratory tract upon infection with *B. bronchiseptica* have been reported so far. In accordance with the literature, we found that *B. bronchiseptica* infection led to a relatively fast pro-inflammatory cytokine response. In contrast, *S. suis* infection barely induced the expression and secretion of pro-inflammatory cytokines in ALI cultures or PCLS. Similar results were described for *S. suis* infection of newborn pig tracheal epithelial (NPTr) cells, as *IL-1α, IL-6, IL-8* (*CXCL8*), and *VCAM1* were not expressed (14, 36). Interestingly, another study reported no induction of *TNFα* but intermediate expression of *IL-6* and high expression of *IL-8* (*CXCL8*) upon infection of the same cells (37). All studies used highly virulent serotype 2 strains (*S. suis* 31533 or *S. suis* P1/7) but different bacterial incubation times. Thus, induction of *IL-6* and *IL-8* (*CXCL8*) expression by *S. suis* might be time-dependent and only activated after prolonged infection, which we also observed in our PCLS infection model. A difference in the temporal dynamics of the host’s immune response was also reported in our previous study, in which we showed that expression of pro- and anti-inflammatory cytokines peaked early during infection of PCLS with a non-virulent and a moderately virulent *S. suis* strain, whereas gene expression peaked much later (24 h post-infection) when infected with the highly virulent strain 10 (the same strain as used in the herein study) (20). Notably, although expression of *CXCL8* increased after prolonged infection, CXCL8 levels in the supernatant of PCLS mono- and co-infected with *S. suis* were significantly reduced. This might be explained by cleavage of CXCL8 by a serine protease of *S. suis* as described by Vanier et al. (38). Co-infection of PCLS with *B. bronchiseptica* and *S. suis* resulted in a higher expression of *IL-1α*, *IL-6*, and *CXCL8* during early infection compared to the levels after mono-infection with either of the pathogens. Similar results were described for co-infection of NPTr cells with SIV (H1N1) and *S. suis* (36, 37), *Glasserella parasuis* and *S. suis* (14), or *Mycoplasma spp*. and *S. suis* (13), whereby the authors called it an additive rather than a synergistic effect. Although these and our results were obtained from *in vitro* infection experiments, one can hypothesize that the enhanced inflammatory immune response might contribute to aggravated streptococcal disease, as an immune system that is already dealing with one infection may not be as resilient to a secondary infection. In *in vivo* infection experiments, Hau et al. did not observe a higher incidence of *S. suis* disease in pigs pre-infected with *B. bronchiseptica*. Nevertheless, the authors stated that *B. bronchiseptica* pre-infection might enhance streptococcal disease when pigs are additionally immunocompromised or stressed (28).

It has to be noted that, with regard to the host’s immune response, PCLS mimic the *in vivo* situation more closely than ALI cultures, as PCLS contain several resident immune cells, such as antigen-presenting cells, macrophages, and T cells (23, 24), and show characteristic responses to pro-inflammatory stimuli (39), whereas ALI cultures consist of epithelial cells only. A recent study on the anti-pertussis response of human airway epithelium revealed a dose-dependent secretion of IL-6 and CXCL8 after infection with *B. pertussis* only, while an additional treatment with IFN-γ, IL-1β, and TNF (cytokines that are secreted by immune cells in response to *B. pertussis*) resulted in secretion of several chemokines, indicating that immune cells are required for an effective immune response (40). Nevertheless, epithelial cells play a critical role in the innate immune response through the secretion of mucin, antimicrobial peptides, and reactive oxygen species, as well as cytokines and chemokines, which *in vivo* recruit and activate other immune cells (41, 42). Thus, both models complement each other perfectly and are most suitable to investigate host-pathogen as well as pathogen-pathogen interactions. Another point that should be considered when working with primary cell cultures is that both immune cells and epithelial cells may already have been activated by previous infections or other harmful substances during the pig’s lifetime, even if the pigs were apparently healthy at the time of slaughter. Although this more closely reflects the *in vivo* situation where several pathogens might colonize the pig’s respiratory tract sequentially or even simultaneously, or airways might be already impaired by bad air conditions, it must be taken into account when interpreting *in vitro* data from primary cell cultures.

### Conclusion

In this study, we analyzed the role of DNT during mono- and co-infections of the porcine respiratory tract *in vitro* and found no evidence that DNT contributes to colonization capacity or cytotoxic activity of *B. bronchiseptica,* nor does it facilitate co-infection with *S. suis* in any way.

Furthermore, we are the first to describe the pro-inflammatory cytokine response in the porcine respiratory epithelial cells *in vitro* upon mono-infection with *B. bronchiseptica* and co-infection with *S. suis. B. bronchiseptica* activated an early pro-inflammatory immune response, independent of DNT, whereas expression and secretion of cytokines peaked late upon infection with *S. suis*. Co-infection with both pathogens resulted in increased levels of the herein analyzed cytokines, suggesting that an enhanced inflammatory response might aggravate streptococcal disease.

Nevertheless, further investigations are necessary to analyze the complex interactions between the two pathogens as well as their interactions with host cells and the host’s immune system in more detail. The porcine PCLS model, as well as porcine respiratory epithelial cells differentiated under air-liquid interface conditions (ALI cultures), represents excellent *in vitro* systems to study these interactions.

## Data Availability

The authors confirm that all data supporting the findings of this study are available within the article or in the Zenodo data repository (https://doi.org/10.5281/zenodo.18400453). All supplemental figures and tables are also available in the Zenodo data repository (https://doi.org/10.5281/zenodo.18400453). Raw sequence data of *Bordetella bronchiseptica* KM24 whole genome sequencing have been deposited in NCBI Sequence Read Archive under accession number PRJNA1214268 and the genome of KM24 is available in NCBI GenBank under accession number CP181209.

## References

[1] Magyar T, Lax AJ. 2002. Atrophic rhinitis. *In* Brogden KA, Guthmiller JM (ed), Polymicrobial diseases. ASM Press, Washington (DC). 21735561

[2] Walker KE, Weiss AA. 1994. Characterization of the dermonecrotic toxin in members of the genus Bordetella. Infect Immun 62:3817–3828. doi:10.1128/iai.62.9.3817-3828.1994 8063398 PMC303036

[3] Teruya S, Hiramatsu Y, Nakamura K, Fukui-Miyazaki A, Tsukamoto K, Shinoda N, Motooka D, Nakamura S, Ishigaki K, Shinzawa N, Nishida T, Sugihara F, Maeda Y, Horiguchi Y. 2020. Bordetella dermonecrotic toxin is a neurotropic virulence factor that uses Ca_V_3.1 as the cell surface receptor. mBio 11:e03146-19. doi:10.1128/mBio.03146-19 32209694 PMC7157530

[4] Park J, Zhang Y, Buboltz AM, Zhang X, Schuster SC, Ahuja U, Liu M, Miller JF, Sebaihia M, Bentley SD, Parkhill J, Harvill ET. 2012. Comparative genomics of the classical Bordetella subspecies: the evolution and exchange of virulence-associated diversity amongst closely related pathogens. BMC Genomics 13:545. doi:10.1186/1471-2164-13-545 23051057 PMC3533505

[5] Horiguchi Y, Senda T, Sugimoto N, Katahira J, Matsuda M. 1995. Bordetella bronchiseptica dermonecrotizing toxin stimulates assembly of actin stress fibers and focal adhesions by modifying the small GTP-binding protein rho. J Cell Sci 108:3243–3251. doi:10.1242/jcs.108.10.3243 7593285

[6] Horiguchi Y, Nakai T, Kume K. 1991. Effects of Bordetella bronchiseptica dermonecrotic toxin on the structure and function of osteoblastic clone MC3T3-e1 cells. Infect Immun 59:1112–1116. doi:10.1128/iai.59.3.1112-1116.1991 1997414 PMC258375

[7] Stanek O, Linhartova I, Holubova J, Bumba L, Gardian Z, Malandra A, Bockova B, Teruya S, Horiguchi Y, Osicka R, Sebo P. 2020. Production of highly active recombinant dermonecrotic toxin of Bordetella pertussis. Toxins (Basel) 12:596. doi:10.3390/toxins12090596 32942577 PMC7551409

[8] Vötsch D, Willenborg M, Baumgärtner W, Rohde M, Valentin-Weigand P. 2021. Bordetella bronchiseptica promotes adherence, colonization, and cytotoxicity of Streptococcus suis in a porcine precision-cut lung slice model. Virulence 12:84–95. doi:10.1080/21505594.2020.1858604 33372837 PMC7781633

[9] Meng F, Tong J, Vötsch D, Peng J-Y, Cai X, Willenborg M, Herrler G, Wu N-H, Valentin-Weigand P. 2019. Viral coinfection replaces effects of suilysin on Streptococcus suis adherence to and invasion of respiratory epithelial cells grown under air-liquid interface conditions. Infect Immun 87:e00350-19. doi:10.1128/IAI.00350-19 31138613 PMC6652749

[10] Meng F, Wu NH, Nerlich A, Herrler G, Valentin-Weigand P, Seitz M. 2015. Dynamic virus-bacterium interactions in a porcine precision-cut lung slice coinfection model: swine influenza virus paves the way for Streptococcus suis infection in a two-step process. Infect Immun 83:2806–2815. doi:10.1128/IAI.00171-15 25916988 PMC4468551

[11] Brockmeier SL, Register KB, Magyar T, Lax AJ, Pullinger GD, Kunkle RA. 2002. Role of the dermonecrotic toxin of Bordetella bronchiseptica in the pathogenesis of respiratory disease in swine. Infect Immun 70:481–490. doi:10.1128/IAI.70.2.481-490.2002 11796573 PMC127710

[12] Lijek RS, Weiser JN. 2012. Co-infection subverts mucosal immunity in the upper respiratory tract. Curr Opin Immunol 24:417–423. doi:10.1016/j.coi.2012.05.005 22658762 PMC3423578

[13] Pageaut H, Lacouture S, Lehoux M, Marois-Créhan C, Segura M, Gottschalk M. 2023. Interactions of Mycoplasma hyopneumoniae and/or Mycoplasma hyorhinis with Streptococcus suis serotype 2 using in vitro co-infection models with swine cells. Pathogens 12:866. doi:10.3390/pathogens12070866 37513713 PMC10383509

[14] Mathieu-Denoncourt A, Letendre C, Auger JP, Segura M, Aragon V, Lacouture S, Gottschalk M. 2018. Limited interactions between Streptococcus suis and Haemophilus parasuis in in vitro co-infection studies. Pathogens 7:7. doi:10.3390/pathogens7010007 29316613 PMC5874733

[15] Ratner AJ, Lysenko ES, Paul MN, Weiser JN. 2005. Synergistic proinflammatory responses induced by polymicrobial colonization of epithelial surfaces. Proc Natl Acad Sci USA 102:3429–3434. doi:10.1073/pnas.0500599102 15728393 PMC552945

[16] Genna VG, Adamo D, Galaverni G, Lepore F, Boraldi F, Quaglino D, Lococo F, Pellegrini G. 2023. Validation of airway porcine epithelial cells as an alternative to human in vitro preclinical studies. Sci Rep 13:16290. doi:10.1038/s41598-023-43284-7 37770485 PMC10539525

[17] Viana F, O’Kane CM, Schroeder GN. 2022. Precision-cut lung slices: a powerful ex vivo model to investigate respiratory infectious diseases. Mol Microbiol 117:578–588. doi:10.1111/mmi.14817 34570407 PMC9298270

[18] Meng F, Wu NH, Seitz M, Herrler G, Valentin-Weigand P. 2016. Efficient suilysin-mediated invasion and apoptosis in porcine respiratory epithelial cells after streptococcal infection under air-liquid interface conditions. Sci Rep 6:26748. doi:10.1038/srep26748 27229328 PMC4882623

[19] Voetsch D. 2020. Novel insights into the role of the Streptococcus suis toxin suilysin in mono- and coinfections of different porcine respiratory epithelial cell culture systems. University of Veterinary Medicine Hannover, Foundation. Available from: https://elib.tiho-hannover.de/receive/tiho_mods_00001257?q=voetsch

[20] Weldearegay YB, Brogaard L, Nerlich A, Schaaf D, Heegaard PMH, Valentin-Weigand P. 2023. Transcriptional host responses to infection with Streptococcus suis in a porcine precision-cut lung slice model: between-strain differences suggest association with virulence potential. Pathogens 13:4. doi:10.3390/pathogens13010004 38276150 PMC10820225

[21] Livak KJ, Schmittgen TD. 2001. Analysis of relative gene expression data using real-time quantitative PCR and the 2^−ΔΔC_T_^ method. Methods 25:402–408. doi:10.1006/meth.2001.1262 11846609

[22] Pullinger GD, Adams TE, Mullan PB, Garrod TI, Lax AJ. 1996. Cloning, expression, and molecular characterization of the dermonecrotic toxin gene of Bordetella spp. Infect Immun 64:4163–4171. doi:10.1128/iai.64.10.4163-4171.1996 8926084 PMC174352

[23] Lyons-Cohen MR, Thomas SY, Cook DN, Nakano H. 2017. Precision-cut mouse lung slices to visualize live pulmonary dendritic cells. J Vis Exp:55465. doi:10.3791/55465 PMC5512285 28448013

[24] Niehof M, Hildebrandt T, Danov O, Arndt K, Koschmann J, Dahlmann F, Hansen T, Sewald K. 2017. RNA isolation from precision-cut lung slices (PCLS) from different species. BMC Res Notes 10:121. doi:10.1186/s13104-017-2447-6 28274266 PMC5343379

[25] Cao Q, Wei W, Wang H, Wang Z, Lv Y, Dai M, Tan C, Chen H, Wang X. 2021. Cleavage of E-cadherin by porcine respiratory bacterial pathogens facilitates airway epithelial barrier disruption and bacterial paracellular transmigration. Virulence 12:2296–2313. doi:10.1080/21505594.2021.1966996 34482810 PMC8425755

[26] Hasan S, Kulkarni NN, Asbjarnarson A, Linhartova I, Osicka R, Sebo P, Gudmundsson GH. 2018. Bordetella pertussis adenylate cyclase toxin disrupts functional integrity of bronchial epithelial layers. Infect Immun 86:e00445-17. doi:10.1128/IAI.00445-17 29203545 PMC5820963

[27] Kessie DK, Lodes N, Oberwinkler H, Goldman WE, Walles T, Steinke M, Gross R. 2020. Activity of tracheal cytotoxin of Bordetella pertussis in a human tracheobronchial 3D tissue model. Front Cell Infect Microbiol 10:614994. doi:10.3389/fcimb.2020.614994 33585281 PMC7873972

[28] Hau SJ, Nielsen DW, Brockmeier SL. 2023. Prior infection with Bordetella bronchiseptica enhanced colonization but not disease with Streptococcus suis. Vet Microbiol 284:109841. doi:10.1016/j.vetmic.2023.109841 37542929

[29] Goldman WE, Klapper DG, Baseman JB. 1982. Detection, isolation, and analysis of a released Bordetella pertussis product toxic to cultured tracheal cells. Infect Immun 36:782–794. doi:10.1128/iai.36.2.782-794.1982 6177637 PMC351298

[30] Brockmeier SL, Register KB. 2007. Expression of the dermonecrotic toxin by Bordetella bronchiseptica is not necessary for predisposing to infection with toxigenic Pasteurella multocida. Vet Microbiol 125:284–289. doi:10.1016/j.vetmic.2007.05.022 17624695

[31] Harvill ET, Preston A, Cotter PA, Allen AG, Maskell DJ, Miller JF. 2000. Multiple roles for Bordetella lipopolysaccharide molecules during respiratory tract infection. Infect Immun 68:6720–6728. doi:10.1128/IAI.68.12.6720-6728.2000 11083787 PMC97772

[32] van den Akker WM. 1997. Bordetella bronchiseptica has a BvgAS-controlled cytotoxic effect upon interaction with epithelial cells. FEMS Microbiol Lett 156:239–244. doi:10.1111/j.1574-6968.1997.tb12734.x 9513272

[33] Higgins SC, Lavelle EC, McCann C, Keogh B, McNeela E, Byrne P, O’Gorman B, Jarnicki A, McGuirk P, Mills KHG. 2003. Toll-like receptor 4-mediated innate IL-10 activates antigen-specific regulatory T cells and confers resistance to Bordetella pertussis by inhibiting inflammatory pathology. J Immunol 171:3119–3127. doi:10.4049/jimmunol.171.6.3119 12960338

[34] Mann PB, Elder KD, Kennett MJ, Harvill ET. 2004. Toll-like receptor 4-dependent early elicited tumor necrosis factor alpha expression is critical for innate host defense against Bordetella bronchiseptica. Infect Immun 72:6650–6658. doi:10.1128/IAI.72.11.6650-6658.2004 15501798 PMC523027

[35] Bassinet L, Fitting C, Housset B, Cavaillon JM, Guiso N. 2004. Bordetella pertussis adenylate cyclase-hemolysin induces interleukin-6 secretion by human tracheal epithelial cells. Infect Immun 72:5530–5533. doi:10.1128/IAI.72.9.5530-5533.2004 15322060 PMC517437

[36] Dang Y, Lachance C, Wang Y, Gagnon CA, Savard C, Segura M, Grenier D, Gottschalk M. 2014. Transcriptional approach to study porcine tracheal epithelial cells individually or dually infected with swine influenza virus and Streptococcus suis. BMC Vet Res 10:86. doi:10.1186/1746-6148-10-86 24708855 PMC4022123

[37] Wang Y, Gagnon CA, Savard C, Music N, Srednik M, Segura M, Lachance C, Bellehumeur C, Gottschalk M. 2013. Capsular sialic acid of Streptococcus suis serotype 2 binds to swine influenza virus and enhances bacterial interactions with virus-infected tracheal epithelial cells. Infect Immun 81:4498–4508. doi:10.1128/IAI.00818-13 24082069 PMC3837972

[38] Vanier G, Segura M, Lecours MP, Grenier D, Gottschalk M. 2009. Porcine brain microvascular endothelial cell-derived interleukin-8 is first induced and then degraded by Streptococcus suis. Microb Pathog 46:135–143. doi:10.1016/j.micpath.2008.11.004 19100324

[39] Henjakovic M, Sewald K, Switalla S, Kaiser D, Müller M, Veres TZ, Martin C, Uhlig S, Krug N, Braun A. 2008. Ex vivo testing of immune responses in precision-cut lung slices. Toxicol Appl Pharmacol 231:68–76. doi:10.1016/j.taap.2008.04.003 18504053

[40] Kroes MM, Miranda-Bedate A, Jacobi RHJ, van Woudenbergh E, den Hartog G, van Putten JPM, de Wit J, Pinelli E. 2022. Bordetella pertussis-infected innate immune cells drive the anti-pertussis response of human airway epithelium. Sci Rep 12:3622. doi:10.1038/s41598-022-07603-8 35256671 PMC8901624

[41] Vareille M, Kieninger E, Edwards MR, Regamey N. 2011. The airway epithelium: soldier in the fight against respiratory viruses. Clin Microbiol Rev 24:210–229. doi:10.1128/CMR.00014-10 21233513 PMC3021210

[42] Ryu JH, Kim CH, Yoon JH. 2010. Innate immune responses of the airway epithelium. Mol Cells 30:173–183. doi:10.1007/s10059-010-0146-4 20878312

